# Characterization and annotation of *Babesia orientalis* apicoplast genome

**DOI:** 10.1186/s13071-015-1158-x

**Published:** 2015-10-16

**Authors:** Yuan Huang, Lan He, Jinfang Hu, Pei He, Junwei He, Long Yu, Ngabu Malobi, Yanqin Zhou, Bang Shen, JunLong Zhao

**Affiliations:** State Key Laboratory of Agricultural Microbiology, College of Veterinary Medicine, Huazhong Agricultural University, Wuhan, 430070 Hubei P.R. China; Key Laboratory of Animal Epidemical Disease and Infectious Zoonoses, Ministry of Agriculture, Huazhong Agricultural University, Wuhan, 430070 Hubei China

**Keywords:** *Babesia orientalis*, Apicoplast, Comparative analysis, Genome

## Abstract

**Background:**

*Babesia orientalis* is an obligate intraerythrocytic protozoan parasite of the buffalo (*Bubalus bubalis*, Linnaeus, 1758) transmitted by the tick *Rhipicephalus heamaphysaloides*. It is the causative agent of water buffalo babesiosis, one of the most important pathogens of water buffalo in central and southern China. As a member of the phylum Apicomplexa, *B. orientalis* possesses a relatively independent and alga originated organelle the apicoplast. Apicoplasts in other apicomplexa parasites are involved in the biosynthesis of haem, fatty acids, iron-sulphur clusters and isoprenoids. Some of these metabolic pathways were shown to be essential for parasite survival, therefore can serve as potential drug targets.

**Methods:**

30 pairs of primers were designed based on the full genome sequence of *B. orientalis* (unpublished data) and by aligning reported apicoplast genomes of *Babesia bovis* and *Theileria parva*. Conventional PCRs was performed to obtain overlapped fragments to cover the whole apicoplast genome. Then the apicoplast genome of *B.orientalis* was sequenced, assembled and aligned with reported apicoplast genomes of *B. bovis* and *T. parva*. The obtained apicoplast genome was annotated by using Artemis and comparing with published apicomplexan apicoplast genomes. The SSU and LSU nucleotide sequences generated were used in a phylogenetic analysis using the maximum likelihood implemented in MAGE 6.0.

**Results:**

We have obtained and analyzed the complete genome sequence of the *B. orientalis* apicoplast. It consisted of a 33.2 kb circular DNA (78.9 % A + T). The apicoplast genome unidirectionally encodes one large and one small subunit ribosomal RNAs, 24 tRNA genes, 4 DNA-dependent RNA polymerase beta subunits (rpoB, rpoC1, rpoC2a and rpoC2b), 17 ribosomal proteins, one EF-Tu elongation factor, 2 Clp protease chaperones, and 14 hypothetical proteins. In addition, it includes two copies of the clpC gene. The structure and organization of the *B. orientalis* apicoplast genome are most similar to those of the *B. bovis* apicoplast.

**Conclusions:**

This is the first report of the complete sequence of the *B. orientalis* apicoplast genome. This information should be useful in the development of safe and efficient treatment against buffalo babesiosis.

**Electronic supplementary material:**

The online version of this article (doi:10.1186/s13071-015-1158-x) contains supplementary material, which is available to authorized users.

## Background

The protistan phylum Apicomplexa contains many species (e.g., *Eimeria tenella, Plasmodium falciparum*, *Babesia bovis*, *Toxoplasma gondii*, *Cryptosporidium* spp. and *Cyclospora* spp.) that are of great health and economic concerns. Most apicomplexan parasites, with the exception of *Cryptosporidium* spp. and *Gregarina* spp. [1, 2], have a relict, non-photosynthetic plastid called the apicoplast [3, 4]. The apicoplast was acquired by secondary endosymbiosis from a eukaryotic alga (but it is still under debate whether it is from a red or a green alga) [5, 6]. It is involved in critical metabolic pathways such as the synthesis of haem, fatty acids, iron-sulphur clusters and isoprenoids. Some of these metabolic pathways are essential for parasite survival and are considered as potential targets for anti-parasitic drug designs. It was reported that the apicoplast housekeeping machinery, specifically apicoplast DNA replication, transcription and translation, was targeted by ciprofloxacin, thiostrepton and rifampin, respectively [7].

Like mitochondria, the apicoplast possesses its own genome. Thus, the complete apicoplast genomes of several apicomplexan parasites have been characterized, which include *Plasmodium* spp*.*, *Leucocytozoon caulleryi*, *Theileria parva*, *T. gondii*, *E. tenella*, *Cyclospora cayetanensis* and *B. bovis* [8–15]. The entire mitochondria genome of *B. orientalis* has been characterized and the phylogenetic analysis has revealed that *B. orientalis* belong to the *Babesia* clade with *B. bovis* as the closest relationship [16]. However, there is no report on the apicoplast genome of *B. orientalis*.

*B. orientalis* is an intra-erythrocytic protozoan parasite which causes babesiosis with clinical manifestation of fever, anemia, icterus, haemoglobinuria and high mortality in water buffalo. *B. orientalis* differs from *B. bigemina* and *B. bovis* in transmission vectors, morphology, pathogenicity and characteristics of *in vivo* cultivation. It causes significant economic losses in central and south China [17, 18]. Recent work focuses on gene diversity, metabolism process, pathogenicity aspect and identification of new markers to improve the diagnosis and therapy of buffalo babesiosis.

In this study, the full sequence of the *B. orientalis* (Wuhan strain) apicoplast genome was determined, annotated and characterized. This is the first report of the complete nucleotide sequence of the *B. orientalis* apicoplast genome. The data generated contribute to the prevention and control of buffalo babesiosis.

## Methods

### Parasites and animal experiments

Blood samples were collected from water buffalo that were experimentally infected with *B. orientalis* in Huazhong Agricultural University [19]. Genomic DNA was extracted using the QIAamp DNA Blood Mini Kit (Qiagen, Hilden, Germany), according to the manufacturer’s instructions.

### Ethics statement

Experimental animals were housed, fed and given clean drinking water according to the stipulated rules for experimental usage of laboratory animals (the regulation of the administration of affairs concerning experimental animals of P.R. China). All protocols were approved by the Laboratory Animal Research Centre of Hubei province, and the ethical committee of Huazhong Agricultural University (permit number 4200696657).

### Cloning and sequencing of *B. orientalis* apicoplast genome

Partial sequences of the *B. orientalis* apicoplast were initially obtained from a high-throughput whole genome sequencing project (data unpublished). To obtain the full-length sequence of the apicoplast genome and fill the gaps between fragments, we designed primers (Additional file 1: Table S1) based on the apicoplast partial sequences. Conventional PCRs was performed to obtain overlapped fragments to cover the whole apicoplast genome. The PCR amplicons were subjected to sequencing directly on an ABI3700 Autosequencer (Applied Biosystems, Foster City, CA) or cloned into pMD19-T vector (TaKaRa Biotechnology) and sequenced subsequently.

### Sequence analysis and annotation

The Software Artemis [20, 21] was used in the annotation of the *B. orientalis* apicoplast genome. The entire apicoplast genome of *B. orientalis* (GenBank accession no. KT428643) was scanned for potential open reading frames (ORFs). The putative coding regions were conceptually translated and annotated using the published *P. falciparum* and *B. bovis* apicoplast genomes as references. These annotations were further refined by BLAST (http://blast.ncbi.nlm.nih.gov/Blast.cgi) searches against the GenBank database. The small and large subunits of rRNA genes (SSU and LSU rRNA, respectively) were determined by comparing with their counterparts in *T. parva* and *B. bovis*. The tRNA genes were annotated by using tRNAscan-SE server (http://lowelab.ucsc.ucsc.edu/tRNAscan-SE/) with the Mito/Chloroplast model and the Nematode Mito model [22]. The online software CGView (http://stothard.afns.ualberta.ca/cgview_server) [23] was used in generating the genetic maps. The reference genomes used included: AAXT01000007 (*B. bovis*), HM222968 (*Chromera* CCMP3155), fusion of X95275 (IRA) and X95276 (IRB) (*P. falciparum*), NC_004823 (*E. tenella*)*,* AAGK01000009 (*T. parva*) and U87145 (*T. gondii*). Phylogenetic analysis of the SSU and LSU genes was performed using the Maximum Likelihood method implemented in MEGA 6.0 (http://www.megasoftware.net) [24]. The transmembrane domains and functional domains were predicted using TMpred (http://www.ch.embnet.org/software/TMPRED_form.html) [25] and Pfam (http://pfam.sanger.ac.uk/) [26].

## Results and discussion

### Characterization of the circular apicoplast genome of *B. orientalis*

Previous efforts aimed to determine the full genome sequence of *B. orientalis* (unpublished data) has found several contigs containing putative apicoplast genome sequences of this parasite. In order to identify the full length sequence of the apicoplast genome, 30 pairs of PCR specific primers based on the sequence information from the aforementioned contigs were designed to obtain amplicons covering the complete apicoplast genome. Sequencing and assembly of these amplicons have shown that the apicoplast genome of *B. orientalis* is a 33.2 kbp circular DNA with a high A + T content of 78.97 %, similar to that of *B. bovis* (33.3 kbp, A + T% = 78.2 %).

**Fig. 1:**
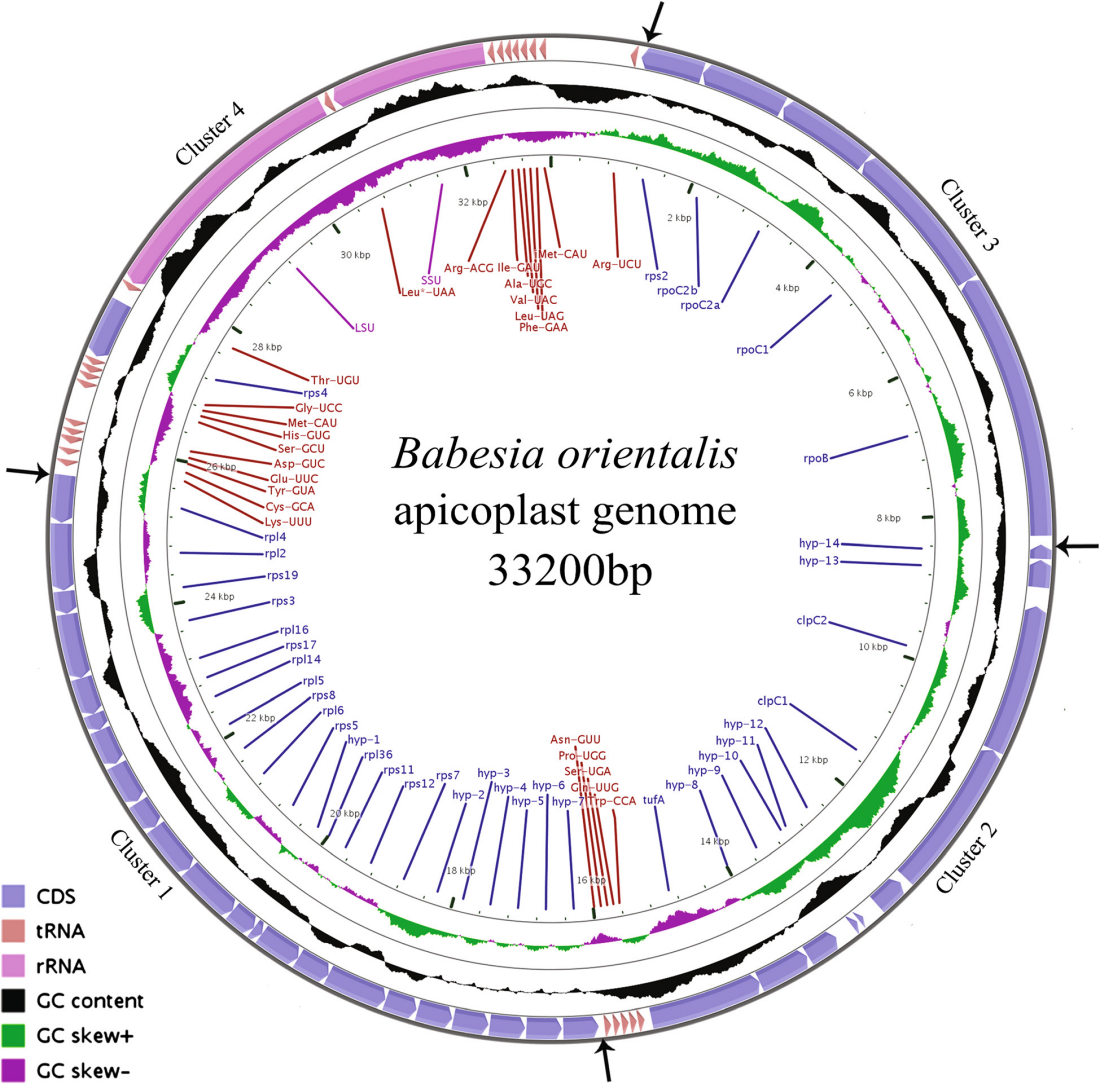
Map of *B. orientalis* (Wuhan strain) apicoplast genome. From outer circle to inner circle:coding sequence (CDs), GC content (%), GC skew and base coordinates. hyp1-14 represent 14 hypothetical protein encoding genes

Bioinformatic analysis indicated that the circular DNA contains 1 LSU rRNA gene, 1 SSU rRNA gene, 38 protein-coding ORFs, and 24 tRNA genes (Table 1). The 38 ORF genes include four DNA-dependent RNA polymerase beta subunits (rpoB, rpoC1, rpoC2a and rpoC2b), 17 ribosomal proteins, and one EF-Tu elongation factor (*Tuf*A), 2 Clp protease chaperone genes, 14 hypothetical proteins ranging in size from 30 (Hyp-11) to 193 amino acids (Hyp-8) (Fig. 1 and Table 1). The *suf*B gene coding for a protein involved in the assembly of iron–sulfur clusters in *Plasmodium falciparum* [14] apicoplast genome was not found in this genome. The content and arrangement of genes in the *B. orientalis* apicoplast genome are more similar to those of *B. bovis* [10] and *T. parva* [11] than those of *P. falciparum* [27] and *T. gondii* [28]. All genes encoded in the apicoplast genome of *B. orientalis* are transcribed in the same orientation and from the same strand (Fig. 1 and Table 1).

**Table 1:** Gene contents of the *B.orientalis* apicoplast genome

Class	Genes
Ribosomal RNA	16S, 23S
Transfer RNA^a,b^	A^UGC^, C^GCA^, D^GUC^, E^UUC^, F^GAA^, G^UCC^, H^GUG^, I^GAU^, K^UUU^, L^UAG^, L^UAA*^
	M^CAU^, M^CAU^, N^GUU^, P^UGG^, Q^UUG^, R^UCU^, R^ACG^, S^GCU^, S^UGA^, T^UGU^, V^UAC^
	W^CCA^, Y^GUA^
Ribosomal proteins	rps2, 3, 4, 5, 7, 8, 11, 12, 17, 19
	rpl2, 4, 5, 6, 14, 16, 36
RNA polymerase	rpoB, rpoC1, rpoC2a, rpoC2b
Other proteins	clpC1, clpC2, tufA
Unassigned ORFs	14 ORFs (hyp1-14)

^a^Single letter amino acid code and anti-codon

^b^Indicating intron-containing genes

The majority of the coding sequences of proteins in the apicoplast genome of *B. orientalis* are not overlapped, except for three genes, which were found to overlap over one to three codons. Twenty-four tRNA genes were identified in the *B. orientalis* apicoplast genome, which are the identical number and type in *T. gondii, T. parva* and *B. bovis*. The tRNA gene Leu^*^-UAA has an intron, and is conserved among these species as well.

The *B. orientalis* apicoplast genome has ribosomal proteins and RNAs very similar to those of other apicomplexans. The genome analysis revealed proteins for the large (7 rpl proteins) small (10 rps proteins) ribosomal subunits. Other ribosomal proteins are encoded by nuclear genome and targeted to the apicoplast according to the analysis of the full genome of *B. orientalis* (unpublished data). Thus ribosomal proteins encoded by the apicoplast and nuclear genomes with 16S and 23S-like rRNA are part of the apiRibosome of the *B. orientalis*. However we did not observe any 5S ribosomal RNA encoding rff gene in the *B. orientalis* apicoplast genome, which indicates that either the apicoplast ribosomes in this species are free of 5S rRNA regulation or the apicoplast can import 5S from the cytoplasm (as shown previously for the mammalian mitochondria [29]). Alternatively, it expresses a highly divergent rff gene. In this perspective, it is noteworthy that although the chloroplast genome of *Chromera* has an rff gene, no rff genes can be observed in the apicoplast genome of any apicomplexan parasite sequences so far.

**Fig. 2:**
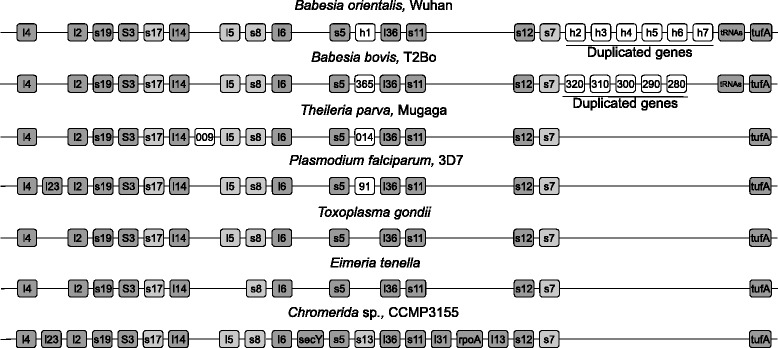
Gene order of cluster 1 in the apicoplast genomes of *B. orientalis* (Wuhan), *B. bovis* (T2Bo), *T. parva* (Mugaga), *P. falciparum* (3D7), *T. gondii*, *E. tenella* and the chloroplast genome of CCMP3155. Light grey boxes indicate highly divergent genes and white boxes correspond to genes restricted to one species

The apicoplast genome of *B. orientalis* has 14 hypothetical coding sequences (hyp1-14). Hyp2-7 are the same ORF but repeat 6 times (Figs. 1 and 2). Some proteins show significant homology to other proteins in available databases but do not have any recognizable functional domains. Other apicomplexan parasites have similar hypothetical proteins in the same genomic regions. Corresponding region in *B. bovis* also exists several same ORF (Hyp 320-280) but repeat only 5 times (Fig. 2). Further work is needed to determine whether these CDs are expressed or are artifacts of annotation.

### *B. orientalis* carries an apicoplast genome similar to *B. bovis*

To determine the synteny of *B. orientalis* apicoplast genome with other apicomplexan parasites, we compared the gene arrangements in four gene clusters (Fig. 1) from the *B. orientalis* apicoplast genome with the same gene clusters found in other apicomplexan parasites as well as the chloroplast genomes of *Chromera* algae [6]. Genes that encode ribosomal proteins and EF-Tu elongation factor are included in cluster 1. As shown in Fig. 2, the apicoplast genome of *B. orientalis* has a gene organization in Cluster 1 region identical to their counterparts in *Babesia bovis* and *Theileria parva*, of which remarkably all lack the rpl23 gene found in *Chromera* and *Plasmodium* species. Additionally the rps13 gene is located between rps5 and rpl36 in *Chromera* sp., whereas that region in most apicoplast genomes, contain an uncharacterized gene or no coding sequence in the case of *T. gondii* and *E. tenella* (Fig. 2). A hypothetical protein (Hyp-1) is found in the same region of *B. orientalis*. Similar to *B. bovis*, there are several putative duplicated CDSs (Hyp2-Hyp7) following rps7 (Fig. 2). In addition, the duplicated CDSs found in *B. orientalis* have high homology to those in *B. bovis*.

**Fig. 3:**
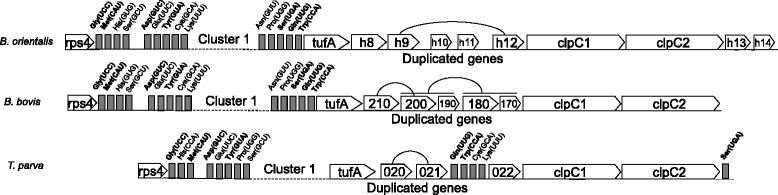
Schematic representation of the genetic organization surrounding cluster 1 in the apicoplast genomes of *B. orientalis* (Wuhan), *B. bovis* (T2Bo), *T. parva* (Mugaga)

**Fig. 4:**
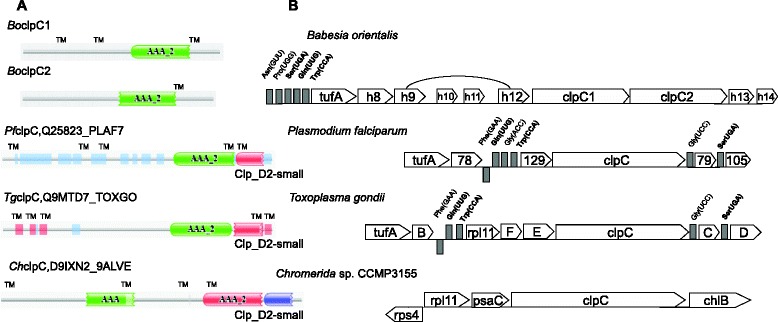
Domain structure of ClpC and gene organization in Cluster 2 of apicoplast genomes from *B. orientalis* and other apicomplexan parasites. **a** Domain structures of ClpC in *B. orientalis*, *P. falciparum*, *T. gondii* and *Chromera* sp. were obtained from Pfam database and TMpred. Two ClpC proteins*,* lack of the N-terminal part, are encoded in *B. orientalis* apicoplast genome. Notably, AAA_2 (ATPase catalytic function) and ClpB_D2-small, as two PfamA domains, exist in ClpC proteins of apicomplexans, but *B. orientalis* has lost the latter. Blue boxes correspond to regions of low complexity. Only *T. gondii* ClpC was predicted by Pfam to contain transmembrane domains (TM). **b** Gene organization in cluster 2 of apicoplast genomes. The tRNA genes are marked in bold as conserved parts of all three apicoplast genomes. Five putative genes, h8 - h12, that lack homologs in other parasites are present in the *B. orientalis* apicoplast genome between tufA and ClpC1

Nine tRNA genes in Cluster 4 of the *B. orientalis* are adjacent to Cluster 1, meanwhile 5 tRNA genes in Cluster 2 are adjacent to Cluster 1 (Fig. 3). One additional tRNA genes for Asn (GUU) is absent in *T. parva* but present between the junction region of Cluster 1 and Cluster 2 in *B. orientalis* and *B. bovis*. Like *T. parva* and *B. bovis*, the junction between Cluster 1 and Cluster 2 in *B. orientalis* has several uncharacterized coding sequences without any clear functions, some of which appear to be identical copies derivated from a duplication process (Boh9 and Boh12). The duplicated CDSs found in *B. orientalis* do not share homology to duplicated sequences present in *B. bovis* and *T. parva* (Fig. 4). Based on these observations, sequences in the area between cluster 1 and cluster 2 is most likely the recombination event region, which probably has played an important role in the evolution of piroplasmida (Figs. 3 and 4).

**Fig. 5:**
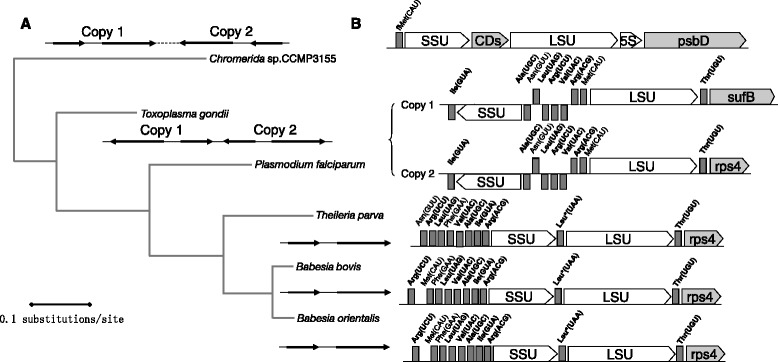
Structure and evolution of the rRNA region in the apicoplast genome of *B. orientalis* and other apicomplexa. **a** Phylogenetic analysis of SSU and LSU rRNA. The tree was built by the maximal-likelihood method with the JTT + F + Γmodel (Bootstrap analysis with 1000 replicates). Genomic structure of rRNA regions in the apicoplast or chloroplast genomes is drawn on top of each branch. **b** Gene order of the rRNA region

ClpC chaperones genes are found in cluster 2 (Fig. 5), have a significant similarity to their orthologs in *B. bovis* and *T. parva*, and are subjected to a duplication process with 2 copies of the AAA_ 2 ATPase domain in *B. orientalis* (Figs. 3 and 4). Two hypothetical proteins (hyp-13 and hyp-14) belong to the area of cluster 2 adjacent to cluster 3 (Figs. 3 and 4).

Similar to *B. bovis*, *P. falciparum* and *T. parva*, we did not observe any rpl11 ribosomal gene in the *B. orientalis* apicoplast genome (Figs. 2 and 5). Thus, *B. orientalis* ribosomes do not need any L11 protein, or an rpl11-like protein encoded in nuclear genome level is actively involved during the process of translation, which is fundamentally different from that showed in *T. gondii* and prokaryotes. Conservation in the order of rpl11-clpC has been reported in *Chromera* and *T. gondii* (Fig. 4), whereas there is a lack of the rpl11 gene in plastid genomes of Aconoidasida, which have shown a rearrangement of the tRNA region adjacent to the clpC gene.

In cluster 3 of the *B. orientalis* apicoplast genome, there are RNA polymerases genes (rpoB, rpoC2a and rpoC2b) and an rps2 gene that encodes S2 ribosomal protein (Fig. 1). Gene content and orientation of Cluster 3 genes in *B. orientalis*, *T. parva* and *B. bovis* is almost the same, suggesting it is a conserved region during the evolution of piroplasmida.

Ribosomal DNA genes are found in Cluster 4, especially a single set of SSU and LSU genes. However, a clear divergence among apicomplexans species regarding gene order and content is observed. In *Chromera*, SSU and LSU separated by a putative coding sequence, have the same orientation. In contrast, the orientation in *Toxoplasma gondii* and *Plasmodium* species is reversed, whereas in *B. orientalis*, *B. bovis* and *T. parva*, the 2 genes are transcribed in the same direction. Like *B. bovis* and *T. parva*, the Leu^*^-UAA tRNA gene with an intron is located between the SSU and LSU genes in *B. orientalis* (Fig. 5). Phylogenetic analysis based on the sequences of SSU and LSU confirmed a close relationship between *B. orientalis* and *B. bovis* (Fig. 5).

Comparative analysis of the apicoplast genomes reveals that rearrangements have occurred over different stages of evolution in the apicoplast establishment, and the loss of genes involved in the photosynthesis physiological pathway has contributed to the apicoplast evolution process. RNA polymerase region inversion, lack of sufB gene, rearrangement of rRNA locus and duplication of clpC gene to form different paralogs are major events in early evolution process of piroplasma organism. The structure modification in the *B. orientalis* apicoplast genome and DNA expansion (duplication of small region), similar to these in *B. bovis*, may be parts of the core apicoplast genome organization in apicomplexans. The only difference of apicoplast genome between *B. orientalis* and *B. bovis* exists in the two regions of duplicated genes in Cluster 1 and Cluster 2 (Figs. 1, 2 and 3).

## Conclusion

In this study, the full *B. orientalis* apicoplast genome was sequenced and annotated. This analysis indicates that the *B. orientalis* apicoplast genome is more similar to that of *B. bovis* and *T. parva* than that of *P. palciparum*, *E. tenella* and *T. gondii.* Complete annotation of the organelle genome indicates that almost all genes identified in the *B. bovis* plastid genome are also present in this parasite. Our studies revealed that this 33.2 kb circular genome encodes machineries involved in gene transcription and translation within this organelle. The genes with metabolic functions are largely absent, although 14 small hypothetical proteins without specified functions need further studies. Our analysis of the apicoplast genome of *B. orientalis* complements our prior annotation of the mitochondria genome of this pathogen [16]. To improve our understanding of the physiological pathway in the apicoplast genome, we are currently trying to identify structural and regulatory proteins that are encoded by the nuclear genome but targeted to the apicoplast. Due to the functional importance of the apicoplast, the information collected from this study contributes to the understanding of metabolism in *B. orientalis* such as the isoprenoid biosynthesis. The potential parasiticidal activities of azithromycin and clindamycin [7] indicate other drug with putative targets in the apicoplast genome can be developed for the treatment of *B. orientalis* infection. Thus, our knowledge of the structural organization of the apicoplast genome of *B. orientalis* will likely facilitate the development of efficient therapy against buffalo babesiosis.

## Additional file

Additional file 1: Table S1. PCR primers used in sequencing the *Babesia orientalis* apicoplast genome. (DOC 67 kb)
