# Supplementary material for: Characterization and annotation of Babesia orientalis apicoplast genome
Source: Parasit Vectors. 2015 Oct 16;8:543. doi: 10.1186/s13071-015-1158-x (PMC4609131; doi:10.1186/s13071-015-1158-x)
Supplement: Additional file 1: Table S1. — PCR primers used in sequencing the Babesia orientalis apicoplast genome. (DOC 67 kb) [file 13071_2015_1158_MOESM1_ESM.doc]

**Additional file 1: Table S1.** PCR primers used in sequencing the *Babesia orientalis* apicoplast genome

| primer | primer sequence (5’→3’) | Position |
| --- | --- | --- |
| Apicoplast-1-F  Apicoplast-1-R | AGATACATAATTATGGGTTAT  TACTAAAAACTAAAATAGATTG | 808-1769 |
| Apicoplast-2-F  Apicoplast-2-R | ACCATCCGGAACCTACCGGTA | 1686-2770 |
|  | CATACTAATTGATAAATATACT |  |
| Apicoplast-3-F | TACAGTAAGTATAGCCCACGC | 2689-3851 |
| Apicoplast-3-R | ATATGGGCCTTACAACAGTTA |  |
| Apicoplast-4-F | ATGTCTTAAAGTTAATGGGCT | 3757-5459 |
| Apicoplast-4-R | ACAAACTTTAATTTCTAAAACA |  |
| Apicoplast-5-F | ATTGTATTTTACACTTACAA | 5384-7036 |
| Apicoplast-5-R | ACCGTTACTAATAGTTATAAT |  |
| Apicoplast-6-F | TCTATAACTTCTATATGTGTA | 6966-8328 |
| Apicoplast-6-R | TAAGATTATTACATCTATTT |  |
| Apicoplast-7-F | ATATGTTTTATAAATTGAAC | 8228-9308 |
| Apicoplast-7-R | TAGACACTATTCTTATTAAGC |  |
| Apicoplast-8-F | ATATTAAGCTTAATAAGAATA | 9281-10702 |
| Apicoplast-8-R | TTACTAACACTAATAATTTTAT |  |
| Apicoplast-9-F | ATATAAATAAGATTTAAATAT | 10615-12413 |
| Apicoplast-9-R | TATAAACTCTATATAAATCAG |  |
| Apicoplast-10-F | ATCGAGATGTATTAGTGATT | 12297-13753 |
| Apicoplast-10-R | TTCAATACATTTGATAATAT |  |
| Apicoplast-11-F | AGATCTAATAAATCTAATAA | 13646-14473 |
| Apicoplast-11-R | ATATAACAGGTACAATAAC |  |
| Apicoplast-12-F | ACTGCGTCAACAATTACATTA | 14380-15585 |
| Apicoplast-12-R | AGAACCTAACTAGCCTTATAG |  |
| Apicoplast-13-F | ATAGTACCTATATTTATATG | 15460-16086 |
| Apicoplast-13-R | TAGTAATAATATAAGTTTTA |  |
| Apicoplast-14-F | ATACAAGTACGACTGTTTTA | 16041-18448 |
| Apicoplast-14-R | AATAATATCCCTTATATTA |  |
| Apicoplast-15-F | ATTCTTAATTAATATAAGGG | 18421-19937 |
| Apicoplast-15-R | ATGAAAAACGAATTAAATAC |  |
| Apicoplast-16-F | ATCTTCTATTGTATTTAATT | 19908-21570 |
| Apicoplast-16-R | ATGATAAAATATTTTAAAAACT |  |
| Apicoplast-17-F | ATCTACTGTATATGTATAAT | 21502-22609 |
| Apicoplast-17-R | ACTATAGTGTCTCATACAA |  |
| Apicoplast-18-F | ACCTGATACCTTTATATTTA | 22555-23572 |
| Apicoplast-18-R | ACCTGTTAATCTTACTGGTA |  |
| Apicoplast-19-F | ACATCCAGAATATTTAACTT | 23530-24542 |
| Apicoplast-19-R | GCAGATAAAGCTAACTGCTA |  |
| Apicoplast-20-F | TCATAGCGGACCCACGTACAA | 24455-25590 |
| Apicoplast-20-R | ATGAATACTTTAGTATTAAATA |  |
| Apicoplast-21-F | TGTATAACAATACTGTAATCA | 25511-26265 |
| Apicoplast-21-R | TCGAGTCCCTTCATTTCTACC |  |
| Apicoplast-22-F | AACTACTTGTATAAAAGTAA | 26208-26969 |
| Apicoplast-22-R | TGTTTGTAACGCCTCTAATACA |  |
| Apicoplast-23-F | AAGCTATAAATAACTATTTA | 26901-27664 |
| Apicoplast-23-R | GGCCCATATTATTCTTATCTG |  |
| Apicoplast-24-F | ACTTATACTTGTTACATACT | 27581-28765 |
| Apicoplast-24-R | GTAAGTTCCGACCTGCATGA |  |
| Apicoplast-25-F | TAAGACAGCGCTCAAATCGT | 28718-29876 |
| Apicoplast-25-R | CTCTGAAATGTATTTAAGTAC |  |
| Apicoplast-26-F | ACTCAGCATGCGTATTAATAG | 29779-30866 |
| Apicoplast-26-R | CTTAACTCCAATAGAATTTAAGT |  |
| Apicoplast-27-F | AGTTAAACTATACTTTTATTA | 30728-31981 |
| Apicoplast-27-R | AGTTCTAAAATAAGTGATTG |  |
| Apicoplast-28-F | CTGCTGGCACAGAGTTAGCC | 31925-32512 |
| Apicoplast-28-R | TTGTACCTTATAATAGTAATA |  |
| Apicoplast-29-F | AAGCTAGGATCTAACTTATAT | 32412-33150 |
| Apicoplast-29-R | ATAAAGCAAAATGGAGTAATA |  |
| Apicoplast-30-F | ATGAGTTATTCTATTACTCCA | 33119-33200(1)-876 |
| Apicoplast-30-R | AATCTTATTAAATTCATTTAT |  |
